# Longitudinal micro-CT as an outcome measure of interstitial lung disease in TNF-transgenic mice

**DOI:** 10.1371/journal.pone.0190678

**Published:** 2018-01-10

**Authors:** Richard D. Bell, Christopher Rudmann, Ronald W. Wood, Edward M. Schwarz, Homaira Rahimi

**Affiliations:** 1 Center for Musculoskeletal Research, University of Rochester School of Medicine and Dentistry, Rochester, New York, United States of America; 2 Department of Pathology and Laboratory Medicine, University of Rochester School of Medicine and Dentistry, Rochester, New York, United States of America; 3 Department of Obstetrics and Gynecology, University of Rochester School of Medicine and Dentistry, Rochester, New York, United States of America; 4 Department of Urology, University of Rochester School of Medicine and Dentistry, Rochester, New York, United States of America; 5 Department of Neuroscience, University of Rochester School of Medicine and Dentistry, Rochester, New York, United States of America; 6 Department of Orthopaedics, University of Rochester School of Medicine and Dentistry, Rochester, New York, United States of America; 7 Department of Pediatrics, University of Rochester School of Medicine and Dentistry, Rochester, New York, United States of America; Forschungszentrum Borstel Leibniz-Zentrum fur Medizin und Biowissenschaften, GERMANY

## Abstract

**Introduction:**

Rheumatoid arthritis associated interstitial lung disease (RA-ILD) is a debilitating condition with poor survival prognosis. High resolution computed tomography (CT) is a common clinical tool to diagnose RA-ILD, and is increasingly being adopted in pre-clinical studies. However, murine models recapitulating RA-ILD are lacking, and CT outcomes for inflammatory lung disease have yet to be formally validated. To address this, we validate μCT outcomes for ILD in the tumor necrosis factor transgenic (TNF-Tg) mouse model of RA.

**Methods:**

Cross sectional μCT was performed on cohorts of male TNF-Tg mice and their WT littermates at 3, 4, 5.5 and 12 months of age (n = 4–6). Lung μCT outcomes measures were determined by segmentation of the μCT datasets to generate Aerated and Tissue volumes. After each scan, lungs were obtained for histopathology and 3 sections stained with hematoxylin and eosin. Automated histomorphometry was performed to quantify the tissue area (nuclei, cytoplasm, and extracellular matrix) and aerated area (white space) within the tissue sections. Spearman’s correlation coefficients were used to evaluate the extent of association between μCT imaging and histopathology endpoints.

**Results:**

TNF-Tg mice had significantly greater tissue volume, total lung volume and mean intensity at all timepoints compared to age matched WT littermates. Histomorphometry also demonstrated a significant increase in tissue area at 3, 4, and 5.5 months of age in TNF-Tg mice. Lung tissue volume was correlated with lung tissue area (ρ = 0.81, p<0.0001), and normalize lung aerated volume was correlated with normalized lung air area (ρ = 0.73, p<0.0001).

**Conclusions:**

We have validated in vivo μCT as a quantitative biomarker of ILD in mice. Further, development of longitudinal measures is critical for dissecting pathologic progression of ILD, and μCT is a useful non-invasive method to study lung inflammation in the TNF-Tg mouse model.

## Introduction

Rheumatoid arthritis associated interstitial lung disease (RA-ILD) is a severe inflammatory condition that develops in 8–15% of all RA patients, and has a median survival expectancy of 2.6 years once diagnosed [1, 2]. RA-ILD has several subtypes including usual interstitial pneumonitis (UIP), non-specific interstitial pneumonitis (NSIP), lymphocytic interstitial pneumonitis (LIP), and desquamative interstitial pneumonitis (DIP). These subtypes can be generally classified into two major categories, fibrotic (UIP and fibrotic NSIP) and cellular based (cellular NSIP, LIP and DIP)[3, 4].

Clinical diagnosis and assessment of RA-ILD typically relies on the use of high resolution computed tomography (HRCT). This imaging modality permits determination of disease severity and progression over time in the clinic, and similar development of in vivo imaging modalities is essential for studying pathogenesis of RA-ILD in animal models. Just as HRCT has become the standard of care for imaging the lung in the clinic over the last decade[5], CT technologies have been adopted for small animal lung disease models[6, 7]. Despite recent advances however, the lung CT literature is limited to a few disease models, primarily used for assessment of cancer models or fibrotic lung disease, not an inflammatory cell driven lung diseases[6, 8].

The tumor necrosis factor transgenic (TNF-Tg) mouse is a well-established model of RA[9, 10]. Recently, investigators have shown that TNF-Tg mice develop a lymphocytic lung disease with significant infiltrates in the perivascular and peribronchiolar space [11–13]. However, course of disease and therapeutic intervention studies in this model have yet to be performed, largely due to the absence of validated in vivo longitudinal outcome measures. To address this major limitation in the field, we aimed to adopt the in vivo μCT method developed for bleomycin induced lung fibrosis[14] to assess the RA-ILD pathology in TNF-Tg mice, and to subsequently validate the μCT outcome measure with histomorphometry. We found a strong correlation between the lung tissue volume measured by μCT and histologic tissue area as well as between lung aerated volume measured by μCT, and lung air area determined by histomorphometry. Thus, μCT imaging can be utilized with confidence as a quantitative longitudinal biomarker of ILD in mice.

## Materials and methods

### Animals

The University of Rochester Medical Center University Committee on Animal Resources (UCAR) has approved of all animal work conducted herein. Anesthesia and Euthanasia were performed in accordance with our approved protocol. Briefly, 1.5–3% Isoflurane was used to anesthetize for all in vivo procedures and 300–360 mg/kg + xylazine 30–40 mg/kg IP was used to overdose the mice followed by cervical dislocation. The 3647 line of the TNF-Tg mouse was obtained originally from Dr. George Kollias[10, 15], and mice were compared to their non-transgenic wildtype (WT) littermates on a C57BL/6 background. Male mice at 3, 4, 5.5 and 12 months of age were imaged and sacrificed for histology (n = 4–6, number of replicates shown on each graph).

### Micro-CT imaging procedure and analysis

**Fig 1:**
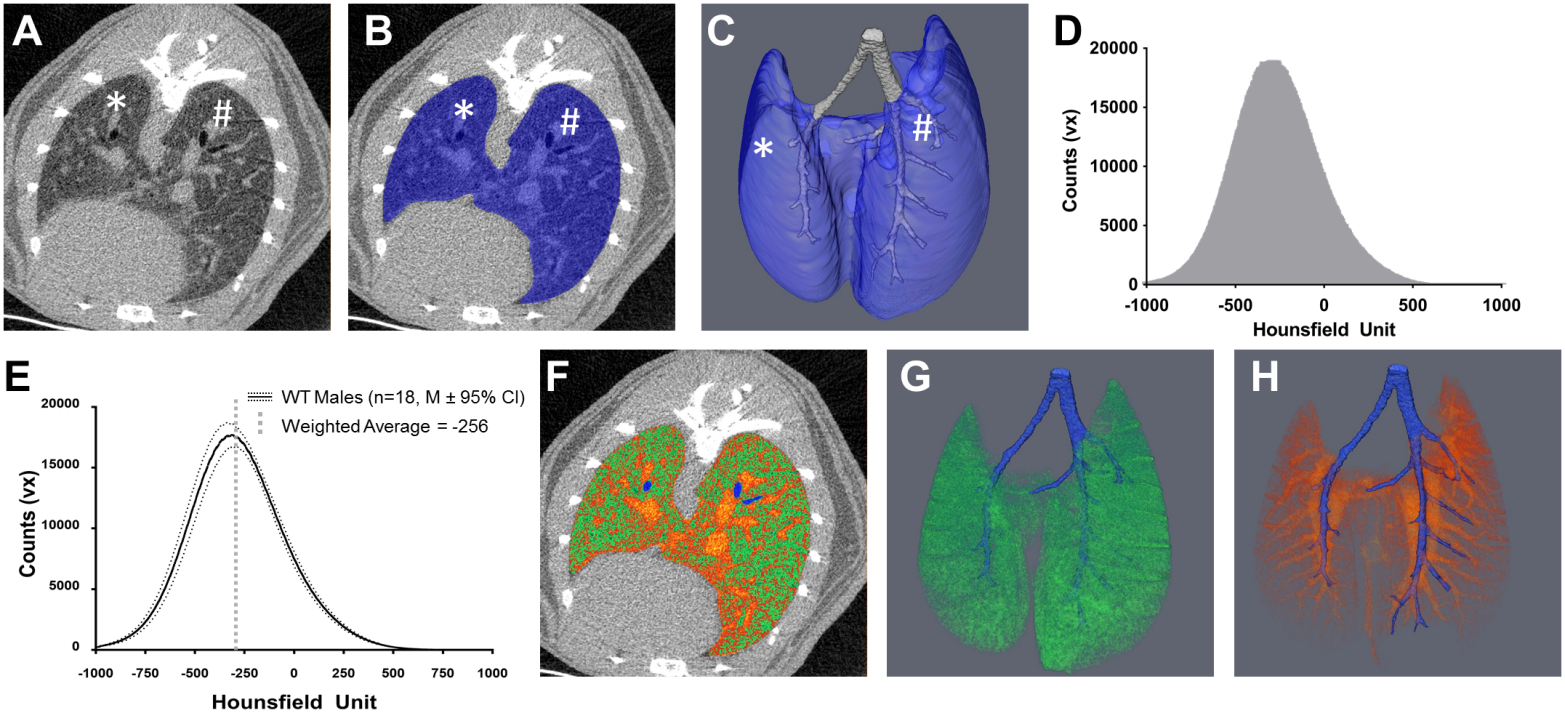
In vivo micro-CT quantification of aerated and tissue lung volume. **A)** A 2D representative μCT transverse, inferior cross section of a WT lung at cervical vertebra 8 shows the margin of the lung inside of the abdominal cavity. **B)** This margin is then used with semi-automated tools in Amira to segment the lung (Blue overlay) and a 3D reconstruction can be generated (**C**). The lung is further segmented into the conducting airway (Grey, **C**) and into the left (*) and right (#) lobes for more specific analysis (**A-C**). The raw intensity values from either the whole lung segment or a smaller portion are extracted to generate intensity histograms (**D**). To delineate aerated lung volume from tissue lung volume a thresholding operation must be developed. The most abundant volume in a healthy lung is the interface between air and the alveolar epithelium. As measured by μCT, the most abundant density in 3–12 month old WT mice is -256 HU (**E**). Using this threshold, aerated and tissue lung volume can be quantified as shown in a representative 2D overlay (**F**, Green = Aerated Volume, Orange-Red = Tissue Volume, Blue = Conducting Airway) and 3D reconstructions (**G**, Aerated Volume; **H**, Tissue Volume, Blue = Conducting Airway). Note the distinct bronchiole and arteriole structures in **G** and **H**.

Mice were anesthetized with 1.5% isoflurane and inserted prone into the imaging tube of a vivaCT 40 μCT (Scanco Medical, Brüttisellen, Switzerland). A pressure pad pillow (RX110, Biopac Systems) was placed underneath the abdomen of the animal and attached to a blood pressure transducer (BPS-BTA and SDAQ, Vernier, Beaverton OR) to detect movement of the chest to gate the μCT imaging to the expiratory pause in breathing. We developed a nonpredictive LabVIEW program (National Instruments, Austin, Texas, USA) that reliably detected the respiratory cycle, and used it to inhibit scanning underway. The μCT scanning parameters were: 1000 projections over 180°, 300 milliseconds integration time, 45 kVp, and 176 micro-amp current. DICOMs at 35 μm^3^ isotropic voxel resolution were imported to Amira (FEI, Hillsboro, Oregon, USA) for semi-automated segmentation, assuming that the lung is a smooth, non-porous organ primarily composed of less dense tissue. The “magic wand” tool, a threshold-based voxel connectivity tool, was used with thresholds of -1000 to -150 and an initial seed point in the bronchus was selected. The growing, shrinking, fill holes and smoothing tools were then used to segment the lung from the rest of the abdomen (Fig 1A–1C). To eliminate the conducting airways from the segmentation, the airways were manually segmented, and measured for diameter and a size exclusion on airways >200 μm was performed (Fig 1C). The intensity of each voxel in Hounsfield Units was then extracted to generate an intensity histogram (Fig 1D) used for all subsequent analyses. The weighted average density was calculated for each lung from the following formula, $\overset{-}{x}\;=\;\frac{\sum_{i\;=\;1}^{n}w_{i}*x_{i}}{\sum_{i\;=\;1}^{n}w_{i}}$, where $\overset{-}{x}\;$ is the weighted mean,*w* is the weight (ie. frequency of the histogram bin), *x*_*i*_ is the Hounsfield unit corresponding to the bin, and n is the number of bins (n = 2001, or -1000 HU to 1000 HU). Bin width has been set to 1 HU. A threshold of -256 HU was derived from the weighted averaged lung intensity of all WT mice in order to differentiate aerated lung and tissue lung volume, (Fig 1E). This threshold value represents the density of the interface between the alveolar epithelium and air in the alveoli, given that this interface is the largest volume in the lung, and is similar to the threshold value previously established for the lung[14]. Once the threshold was set, area under the curve was determined for each partition to obtain three outcome measures: total volume, tissue volume, and aerated volume. Tissue and aerated volumes were divided by the total volume to obtained normalized values, as a measure of how each outcome changes within the context of the organ as a whole. To correlate the μCT with the histologic outcomes, the left and right lungs were separated by anatomic features, and the left lung was used for all correlations (* and # in Fig 1A–1C).

The weighted computed tomography dose index (CTDI_w_) was calculated from the following equations, CTDI_w_ = 1/3* CTDI _center_ + 2/3*CTDI _peripheral_, where CTDI _center_ is the CTDI at the center of the stage and CTDI _peripheral_ is the CTDI around the periphery of the stage. The CTDI was calculated from the following equation $CTDI=\;\int_{-50\;mm}^{+50\;mm}\frac{D\left(z\right)}{N*T}dz$, where N is the number of slices, T is the nominal section thickness, and D(z) is the radiation dose measured at the z position. The measured dosage is provided for a given imaging energy. Time, current, product and resolution is provided by Scanco. For scans <40mins at the imaging parameters we used the total dosage is <0.15 Gy. The gating procedure for the vivaCT 40 closes a shutter when gating is on to ensure termination of the beam.

### Histology procedures and analysis

**Fig 2:**
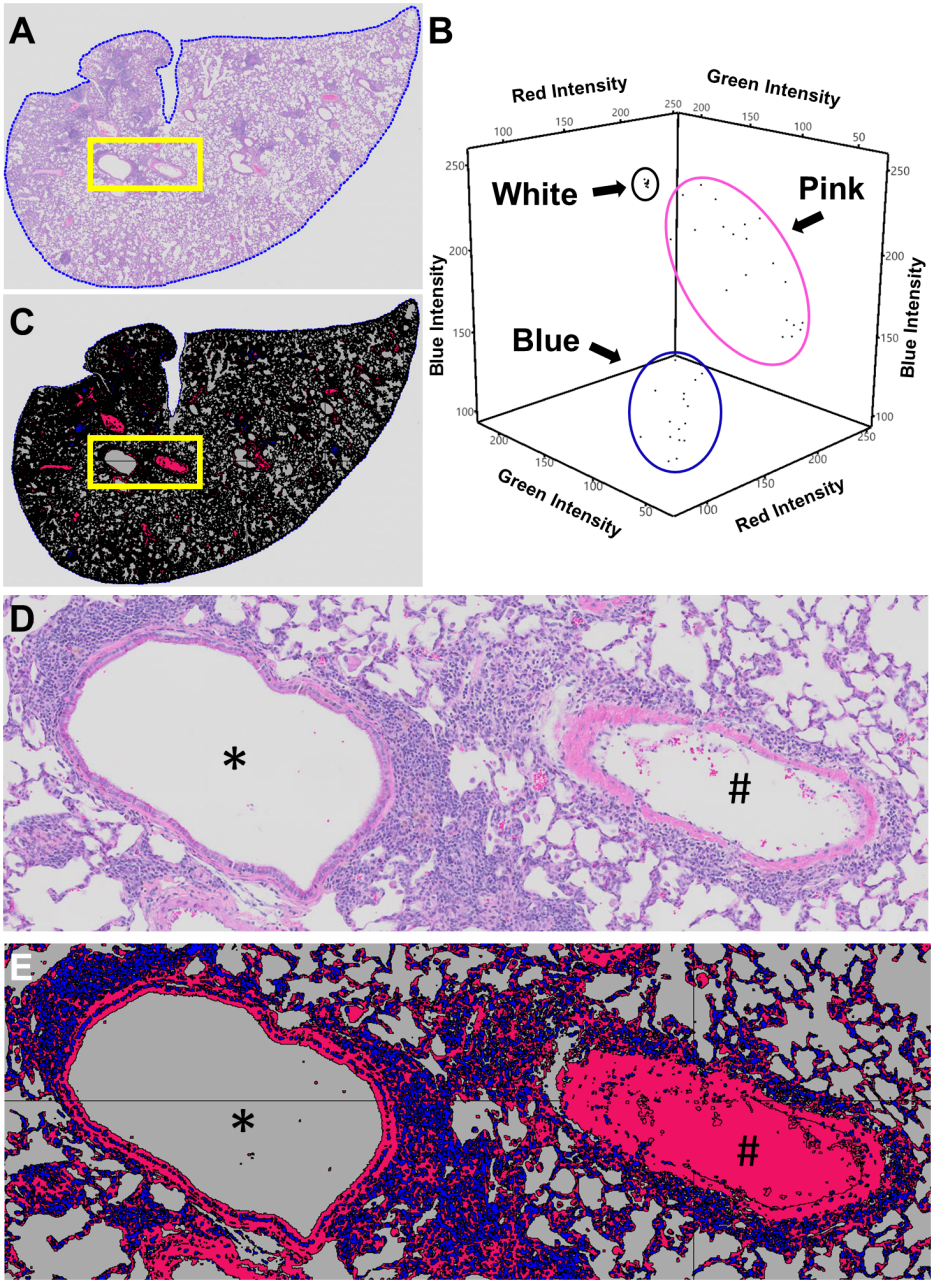
Histomorphometry method for identifying cell nuclei, cytoplasm, extracellular matrix, red blood cells and alveolar space with Visiopharm. To quantify histomorphometric areas, lungs were stained with Hematoxylin and Eosin, whole slides were scanned, imported into Visiopharm and a ROI was drawn around the margin of the lung (**A,** ROI = blue dotted line). A custom application was developed to classify the assorted colors within the ROI of the stained slide into 3 categories (Blue, Pink, and White) by training Visiopharm on the Red/Green/Blue pixel intensity ranges. This occurs through manual selection of a training data set of RGB values for each category and application of a Bayesian discrimination algorithm to determine a set of RGB ranges for each category. An example training data set with >15 pixels for each category is presented in **B** with the intensity of the Red, Blue and Green pixels plotted against each other with each category circled together in 3D space. Note how each category has a distinct range of intensities allowing discrimination of cell nuclei (Blue category) from cytoplasm, ECM and RBCs (Pink category) from alveolar space (White category). Once defined, this classification was applied to every slide in the data set. Representative H and E (**A** and **D**) and classified slides (**C** and **E**) are shown to demonstrate the specificity of our classification method (bronchiole = *; artery = #). For arteries and arterioles that were not filled with RBCs, manual classification was performed to change the space from White to Pink after inspection of the epithelial layer (#, **E**).

At sacrifice, the animals were dissected to expose the thoracic cavity and trachea. A 4–0 silk suture was loosely tied around the trachea and a 10ml syringe fitted with a 25-gauge needle filled with 10% neutral buffered formalin (NBF) was inserted into the trachea. Forceps were used to clamp the needle inside the lumen of the trachea to prevent backflow and instillation of NBF at >20mm H_2_O was performed until there was visual confirmation that all lobes of the lung were fully inflated, at which time the suture was tied tight and the lungs were excised[16, 17]. Then the left lung of each mouse was incubated in 10% formalin for 3 days, processed, embedded in paraffin and sectioned. The lung was sectioned from the dorsal aspect to the ventral aspect in a coronal manner. Three 5 μm thick sections were taken at least 200 μm apart, stained with hematoxylin and eosin, and whole slides were scanned (VS120, Olympus, Tokyo, Japan). These slide scanned images were analyzed with Visiopharm imaging software if there was a minimum of 15 μm^2^ of total lung area (Hoersholm, Demark). A region of interest (ROI) was drawn around the margin of the lung (Fig 2A) and a custom application was developed to detect the colorimetric differences between the shades of blue stain, red/pink stain and the background (white color). These colors were then classified into 3 categories (Blue, Pink, and White) to delineate the difference between the cell nuclei (Blue), cytoplasm, extracellular matrix and red blood cells (Pink) and alveolar space (White, Fig 2B–2E). Because red blood cells are often removed from major arteries during histologic processing, empty arteries were manually reclassified from “white” to “pink” (# in Fig 2D and 2E). These categories were used as independent outcomes, and the blue and pink categories were summated to obtain total lung tissue area. To minimized confounding effects of tissue processing that may decrease soft tissue volume, all histomorphometric categories were also normalized to the total area of the ROI within each slide.

### Statistical analysis

All statistical tests were performed in JMP Pro 13 (SAS, Durham, NC). Data were assed for normality with Shapiro-Wilks tests and data were typically not normally distributed; therefore, normality was restored by rank transformation. Two-way ANOVAs were performed to examine the overall effect of genotype and age with Tukey’s post-hoc for multiple comparisons. When main effects were only observed for genotype, post-hoc *t*-tests with Bonferroni corrections for multiple comparisons (α = 0.05÷4 = 0.0125) were performed within each timepoints. Spearman rank correlation coefficients were computed from the raw, un-transformed data to describe associations between μCT and histology measures.

## Results

### TNF-Tg mice have increased lung tissue volume via in vivo μCT

**Fig 3:**
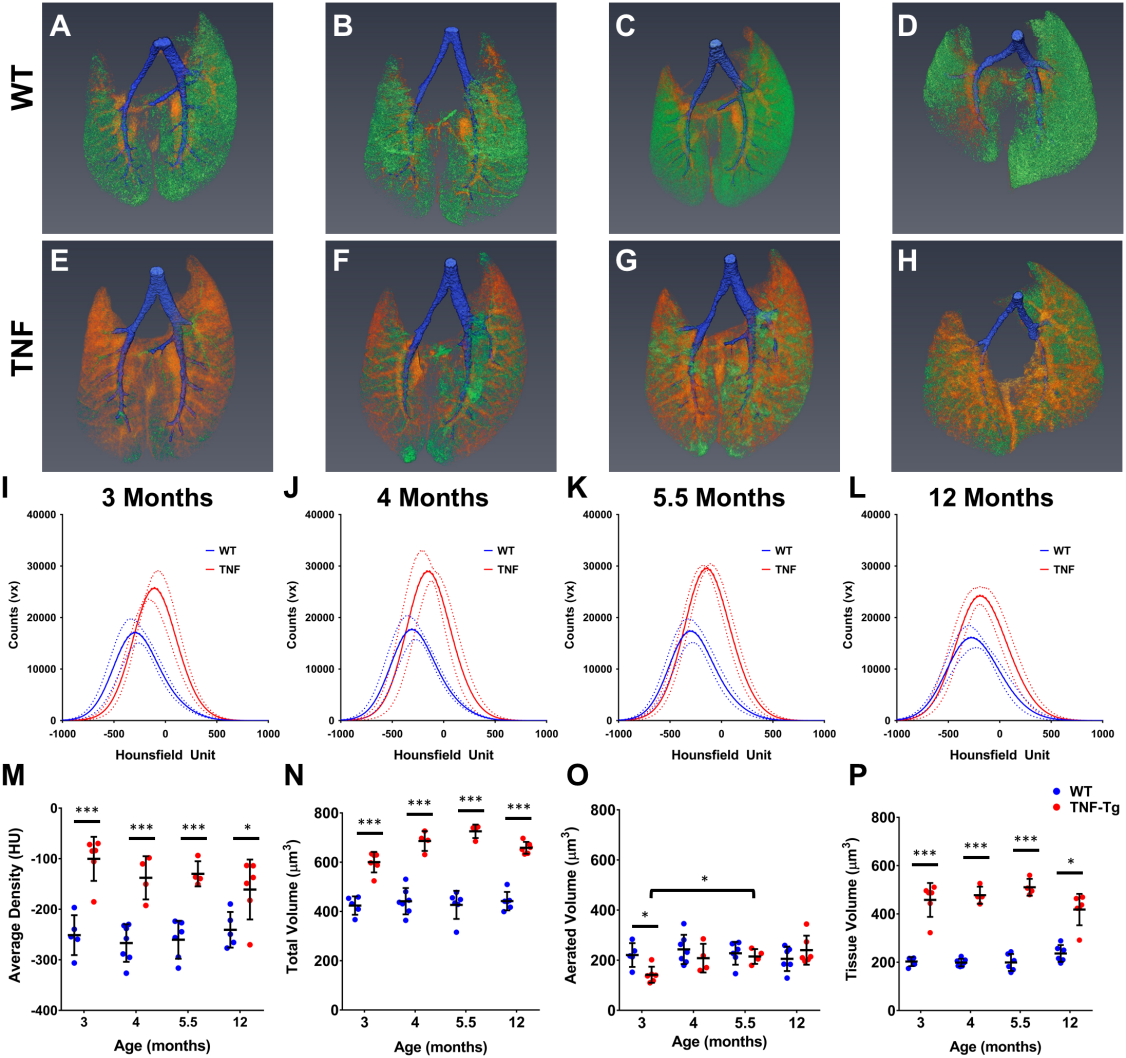
TNF-Tg male mice have increased tissue volume compared to WT littermates measured via μCT. Representative 3D reconstructions of 3 (**A, E**), 4 (**B, F**), 5.5 (**C, G**), and 12 (**D, H**) month old WT and TNF-Tg male mice show a clear increase in tissue volume (Orange-Red) in the TNF-Tg animals at all timepoints while maintaining a similar amount of aerated volume (Green) at all timepoints. Conducting airways that were segmented out of analysis shown in Blue. Histograms of the extracted data from the whole lung segmentation for each timepoint is presented in **I-L** (M ± 95%CI, n = 4–6). TNF-Tg male mice have a statistically significant increase in mean lung intensity (**M),** total lung volume (**N**) and tissue lung volume (**P**) at all timepoints compared WT littermates (*p<0.05, ***p<0.001, M±SD, n = 4–6).

Three dimensional representative reconstructions clearly demonstrate that TNF-Tg mice have greater tissue volume than their WT littermates at every timepoint (Orange-Red in Fig 3A–3H). Tissue density histograms were shifted right and had larger maxima for TNF-Tg lungs compared to WT lungs at every timepoint (Fig 3I–3L). Quantifying the differences between these histograms demonstrates that the weighted average density, total volume and tissue volume were increased at all timepoints for the TNF-Tg lungs compared to their age matched WT counterparts (Fig 3M, 3N and 3P). While the aerated volume in TNF-Tg lungs was decreased at 3 months compared to WT, it recovered to WT levels at 4 months and was significantly different at 5.5 months compare to 3 months (Fig 3O).

### TNF-Tg mice have increased lung tissue area via histomorphometry

**Fig 4:**
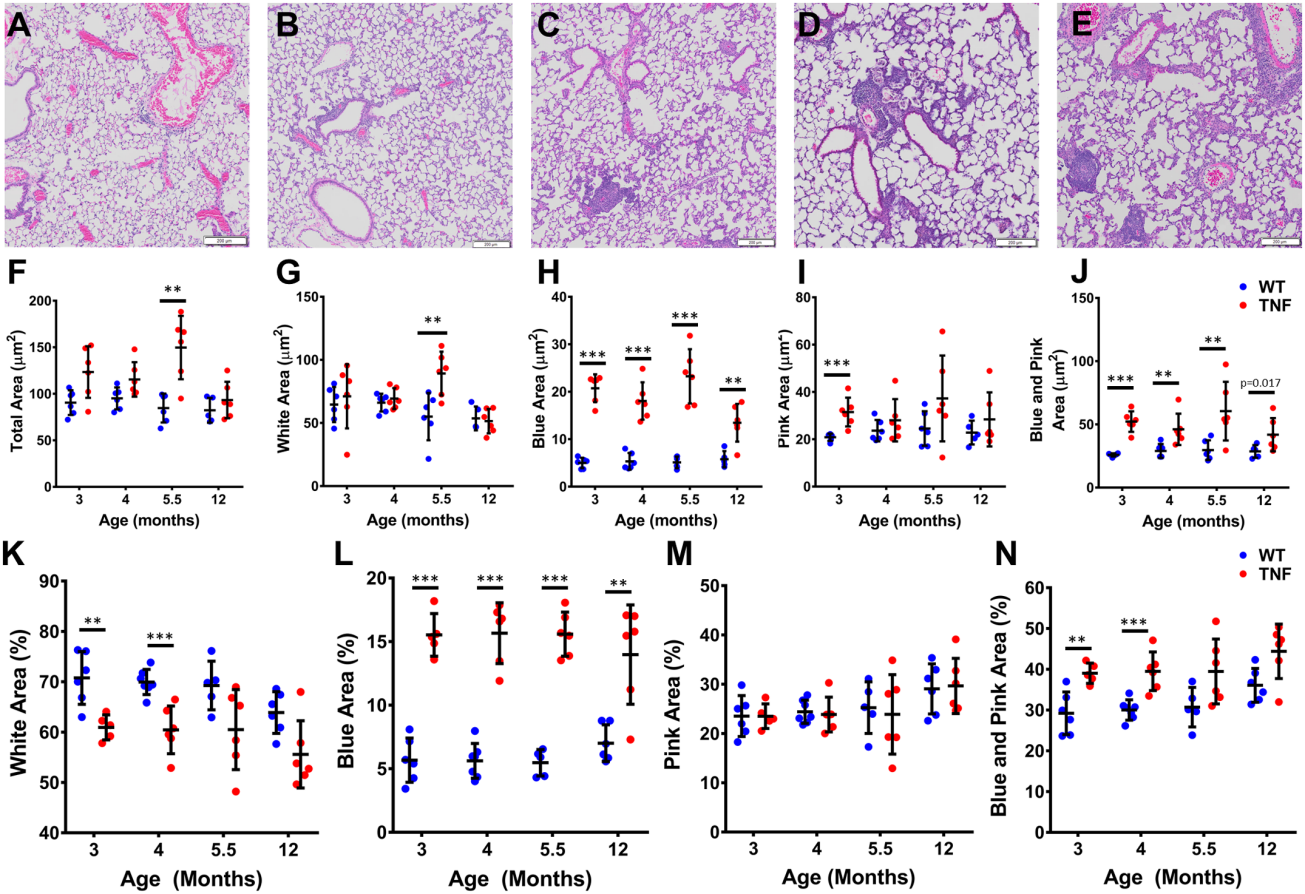
TNF-Tg male mice have greater tissue area compared to WT litter mates measured via histomorphometry. Blue, Pink and White areas were measured on 3 sections >200 μm apart for 3, 4, 5.5, and 12-month-old male TNF-Tg and WT littermates. Representative H and E images from a 12-month WT (**A**), and 3 (**B**), 4 (**C**), 5 (**D**) and 12 (**E**) month TNF-Tg mice show a clear increase in cells in the TNF-Tg mice at all timepoints. Quantifying each histomorphometric category demonstrates a significant increase in Total area at 3 and 5.5 months (**F**), Blue area at all timepoints (**G**) and Blue + Pink at 3, 4, and 5.5 months (**H**) for TNF-Tg mice compared to their WT littermates. Due to the known volumetric changes that occur in the histologic processing solutions (i.e. formalin, ethanols) and the nature of lung tissue being sensitive to changes due to its composition, we normalized all the measurements to the total area (**I**, **J**, **K** and **L**). As expected, percent Blue area (**J**) at all timepoints and percent Blue + Pink (**L**) at 3, 4 and 5.5 months were significantly increased in TNF-Tg mice compared to WT littermates. Interestingly, when normalized to total area, there was a significant decrease in the White area at 3, 4, and 5.5 months in TNF-Tg mice compared to WT littermates (**K;** *p<0.0125, **p<0.01, ***p<0.001, M±SD, n = 4–6).

Representative images of a 12-month WT mouse lung versus lungs from 3, 4, 5.5 and 12-month TNF-Tg mice showed remarkable accumulation of inflammatory cells in the TNF-Tg lungs (Fig 4A–4E). Moreover, we observed an increase in Blue area (Cells) at all timepoints compared to WT littermates, but did not find an increase in Pink area (ECM, Blood, Cytoplasm, ect) at 4, 5.5 or 12 months of age, indicating that the ILD in TNF-Tg mice is primarily inflammatory and not fibrotic (Fig 4H and 4I). When normalized to whole lung area, the percent White area (Air) was significantly decreased at 3, 4, and 5.5 months of age in the TNF-Tg mice (Fig 4K), while the percent Blue area (Cells) remained increased in TNF-Tg mice at all timepoints (Fig 4L).

### Tissue volume via μCT and tissue area via histomorphometry are correlated

**Fig 5:**
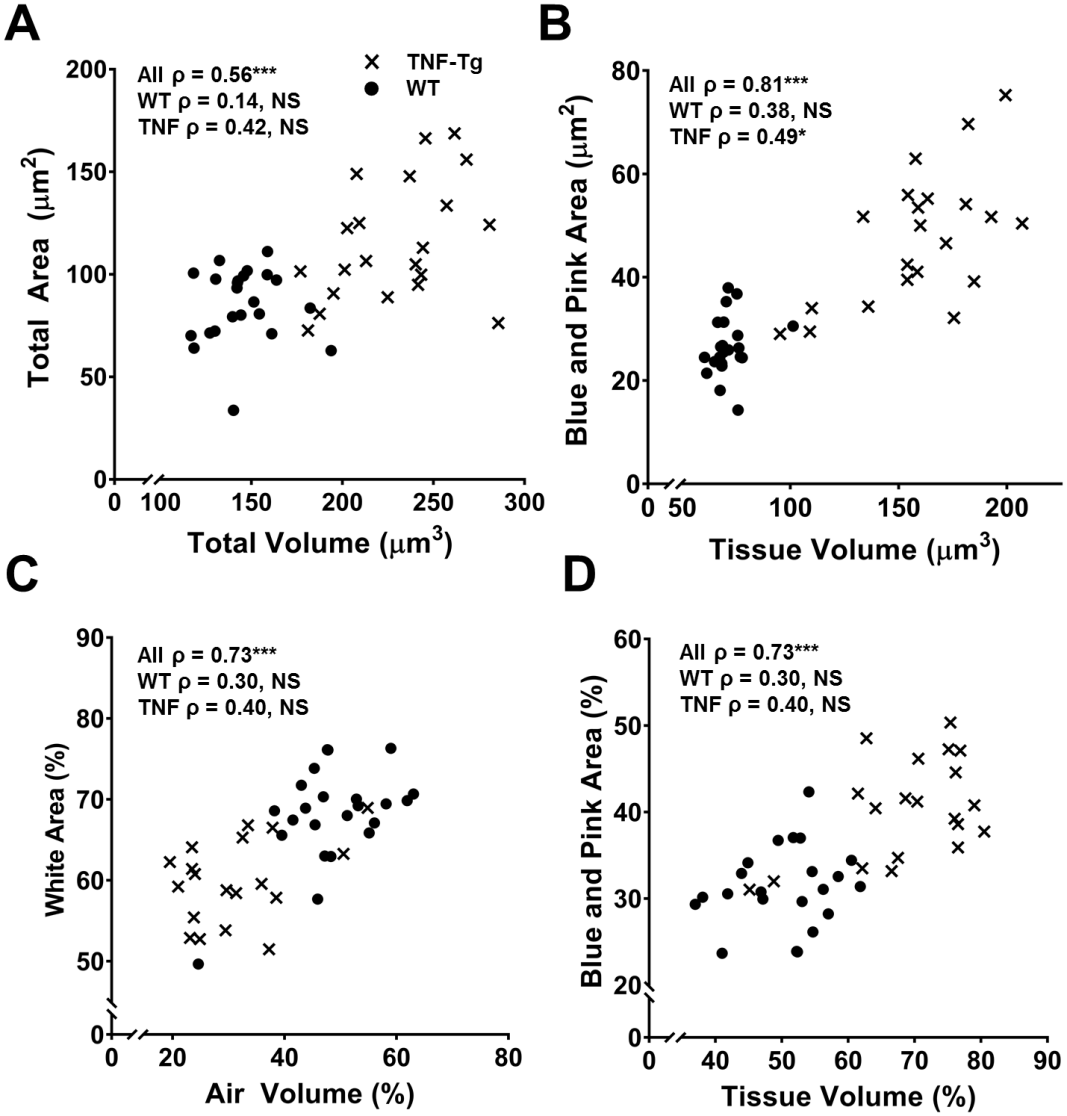
Tissue volume and tissue area (Blue + Pink) are highly correlated. Spearman’s correlations were performed comparing μCT outcome measures and histomorphometry outcome measures. Both total volume and total area (**A**) as well as tissue volume and Blue + Pink (**C**) area were highly correlated with each other. Aerated volume and White area were not correlated (Not Shown); however, when normalized to total volume and area (**C**), there was a significant correlation. Normalized tissue volume and Blue + Pink area remained correlated (**D**). Spearman Rank coefficients are present on each graph for all data points (All ρ) and for each genotype independently (WT ρ, TNF-Tg ρ, X = TNF-Tg, ● = WT, n = 43, NS = not significant, *p<0.05, ***p<0.0001).

Spearman Rank correlation coefficients were computed to describe the association of μCT and histology outcome measures. Total volume by μCT and total area by histomorphometry were highly correlated (ρ = 0.56, p<0.0001, Fig 5A), as were tissue volume and total tissue area (Blue + Pink area) (ρ = 0.82, p<0.0001, Fig 5B), but not aerated volume and White area (ρ = 0.08, p = 0.57). However, when the normalized aerated volume and White area were compared, a significant correlation between these outcomes was found (ρ = 0.72, p<0.0001, Fig 5C). As expected, the correlation between the normalized tissue volume and Blue + Pink area was also significant (ρ = 0.73, p<0.0001, Fig 5D). When the genotypes were analyzed independently, total volume and area were not significantly associated with each other for either genotype (Fig 5A). However, tissue volume and tissue area were associated for TNF-Tg mice independent of their WT littermates (ρ = 0.50, p<0.05, Fig 5B). Both normalized outcomes were not associated independent of genotype (Fig 5C and 5D)

## Discussion

Developing in vivo imaging in small animal disease models is a crucial step towards elucidating the etiology and developing effective interventions for conditions like RA-ILD. Thus, we used μCT methodology to quantify tissue volume, aerated volume and total volume of an inflammatory murine lung disease, and found significant correlations with histomorphometry outcome measures. Importantly, tissue volume and tissue area were highly correlated, suggesting that this μCT outcome measure can be used to assess cellular infiltrate, extracellular matrix deposition, and/or edema in vivo. Prior research has suggested use of similar methods to quantify μCT outcomes in other lung disease models including fibrotic lung disease, emphysema and fungal infections [6, 7, 14]. Our extension of this work with a mouse model of RA-ILD thus expands the utility of in vivo μCT for quantifying lung disease.

While TNF is known to induce pathologic changes in the lung [11, 13], the temporal lung pathology in TNF-Tg mice had not been previously characterized using μCT imaging, nor have histological studies of disease progression been reported. Thus, our finding of significant increases in Blue area (cells) without changes in Pink area confirm previous reports that TNF driven lung disease comprises a largely lymphocytic disease process, but in our model, does not progress to a fibrosing disease [11, 13]. In contrast to our findings, Bawadekar et al observed increased pathology within the lung over time, where as we did not find any temporal differences in lung tissue volume or tissue area with histomorphometry. This may be due to our 35 μm^3^ resolution limiting the ability to identify radiographic features more sensitive to change and that we did not further segment our histomorphometry to investigate specific compartments of the lung (e.g. peribronchiolar, perivascular, septal areas etc.). However, 35 μm^3^ is the highest in vivo resolution we can image at and further automated segmenting of histomorphometry was not possible with our methodology. The finding that our model does not progress to a fibrotic or emphysema-like phenotype contrasts with studies performed in mice overexpressing TNF driven from the surfactant protein C promoter (SP-C/TNF-Tg) restricting expression to the lung [11, 12, 18, 19]. These studies identified an initial lymphocytic alveolitis that progressed to a fibrotic and emphysema like phenotype with collagen deposition in the interstitium, over expanded lungs and enlargement of the alveoli [11, 19]. These structural changes are likely due to TNF induced aberrant expression of remodeling proteins, such as MMPs, TIMPs and CASPs [20]. Interestingly, Aerated volume was decreased at 3 months of age in our TNF-Tg mice compared to WT likely due to smaller aerated volume through post-natal development. This volume increased to WT levels at 4 months suggesting a protective compensation, in marked contrast to acute lung disease models, such as bleomycin induced lung fibrosis, that show an immediate loss of aerated volume with no compensatory recovery across 28 days [6, 7, 14]. However, this increase in aerated lung volume could also be pathologic resembling the emphysema-like phenotype in the SPC/TNF-Tg mice. Further studies investigating lung function and pathophysiology are needed to elucidate these differences between models.

Soft tissues are known to undergo deformation changes during histologic processing, and is an established limitation of histomorphometry. To address this, the American Thoracic Society and the European Respiratory Society have published guidelines regarding the treatment of lung tissue for various outcomes, to minimize histology artifacts between samples [21]. Two guidelines of importance are the instillation pressure and fixative used. Instillation pressure should be >20 mmH_2_O for proper inflation of the lung tissue. Fixatives recommended include either formaldehyde or glutaraldehyde; the former will ensure proper fine tissue fixation and preserve epitopes for immunohistochemistry (IHC) and the latter ensures a strong fixation to stabilize the gross architecture but limits accessibility to epitopes [21]. Due to the interest in further characterization of the lung disease and consistency in future studies in this mouse, we chose neutral buffered formaldehyde to preserve epitopes for IHC. This decision may lead to the potential for volumetric changes between samples and may be a possible reason for the lack of correlation between aerated lung volume and white area. However, the relative relationship between the two outcomes can be assessed by normalizing within each sample to the total volume or area to obtain the relative contribution of each sub-compartment (e.g. aerated volume, tissue volume, blue area, white area). This is demonstrated by the significant correlation of percent aerated volume to percent white area (Fig 5C).

The gold standard for histology outcome measures is to have an expert reader or certified pathologist characterize or categorize pathologic changes within the tissue of interest. While this accepted practice is beneficial, expert graders are susceptible to implicit and explicit bias. Furthermore, since the training and time required to become an expert grader is often prohibitive, automated histomorphometry is an attractive alternative. We therefore developed an automated histomorphometric method to analyze lung ROIs in a cost-effective and unbiased manner that correlated with in vivo μCT outcome measures. It should be noted that this method cannot yet assess specific pathologic findings directly, such as perivascular accumulation of infiltrating immune cells versus arteriolar hypertrophy.

When conducting experiments with ionizing radiation, dosage should be considered due to its known toxic effects, especially in the lungs. In this study, mice received one <40-minute scan calculated to be <0.15 Gy which is well below the known toxic amount of x-ray radiation. When used in longitudinal studies, total radiation exposure must be taken into account, but no adverse effects have been reported from multiple imaging sessions of 0.62 Gy x 13 sessions and 0.813 Gy x 4 sessions [14, 22].

The value used to threshold aerated volume from tissue volume is critically important, as these are the two primary outcomes. We used the weighted average of the WT lung densities to differentiate the two volumes, presuming that the most common density will be the interface between the alveolar epithelium and the air directly adjacent. De Langhe et al reported a threshold of -383 HU that was determined by visual inspection of “lung regions directly above the diaphragm” on μCT scans [14]. We investigated if the results of our study would change if this value was used instead, and all results and interpretations of the data are unchanged. Examination of the raw histograms suggests that many threshold values would confer the outcomes presented herein (Fig 3I and 3L and S1 File). Differences in this thresholding value are to be expected due to a variety of factors including different imaging parameters, μCT machines, or post acquisition processing. However, a unifying logical algorithm can be adopted for cross study comparisons.

One limitation of our μCT method is that the isotropic resolution of our scans (35 μm^3^) prevented evaluation of possibly clinically relevant morphologic features [5]. Higher resolution scans would minimize this issue, but would also significantly increase scan time by a factor of 2–3, thereby increasing radiation dosage and reducing throughput. Additionally, it is unclear whether improved visualization of radiologic characteristics would be informative given that the clinical trajectory is to move away from descriptive imaging and towards more objective parameters[23].

An additional limitation of our μCT method is that we used a semi-automated segmentation method with manual inspection of the whole lung segment that may introduce observer bias; this bias can be overcome using blinding techniques and attending to interobserver reliability measures. Other groups have developed custom automated algorithms to perform segmentation of the air space within lung but these algorithms do not segment the tissue volume[6]. The challenge for automated algorithms is the similarity in density of cellular accumulations within the lung compared to adjacent organs. This is primarily due to CT inability to provide high contrast between the densities of soft tissue, which can likely be overcome with use of a contrast agent.

In summary, we have determined and validated an imaging method for quantifying disease progression in an inflammatory lung model using an established mouse model of RA-ILD. This methodology using μCT to assess inflammatory lung disease progression correlates well with histological disease progression. Therefore, μCT is a useful and cost-effective method for biomarker quantification in longitudinal preclinical studies.

## Supporting information

S1 File Supplemental data. This excel file holds the all supplemental data for this manuscript, including the raw density histograms and their derived outcomes measures “Weighted Average”, “Total Volume”, “Air Volume” and “Tissue Volume” in voxels; as well as histomorphometry measures of “White Area”, “Blue Area”, “Pink Area” and “Total Area” for each histologic level and their derived summed values.
(XLSX)
